# Incidences of Concussion in the United States: A Review of Health Insurance Claims

**DOI:** 10.3390/brainsci16060546

**Published:** 2026-05-22

**Authors:** Alyssa M. Lickfeld, Elizabeth V. Castro, Ava Ferreira, Jazlyn M. Edwards, Alissa Patel, John J. Leddy, Mohammad N. Haider

**Affiliations:** 1 UBMD Department of Orthopaedics and Sports Medicine, Jacobs School of Medicine and Biomedical Sciences, State University of New York at Buffalo, Buffalo, NY 14221, USA; alickfel@buffalo.edu (A.M.L.); ecastro3@buffalo.edu (E.V.C.); avaferreira5@gmail.com (A.F.); jazlyned@buffalo.edu (J.M.E.); patelalissa25@gmail.com (A.P.); leddy@buffalo.edu (J.J.L.); 2 Department of Oral Biology, School of Dental Medicine, State University of New York at Buffalo, Buffalo, NY 14215, USA; 3 Neuroscience PhD Program, Jacobs School of Medicine and Biomedical Sciences, State University of New York at Buffalo, Buffalo, NY 14203, USA

**Keywords:** concussion, post-concussion syndrome, mild traumatic brain injury, incidence, MarketScan

## Abstract

**Background**: Mild traumatic brain injuries (mTBIs) are a significant public health concern in the US. Understanding incidence and demographic patterns is essential for developing targeted prevention and intervention strategies. The most recent study using national healthcare records to examine incidence utilized data from 2016, highlighting the need for updated estimates that reflect current trends. **Methods**: The MarketScan^®^ Database was used for this study which includes person-specific clinical utilization, expenditures, and enrollment across different services. A query for mTBIs (S06.0x.xx) or post-concussion syndrome (F07.89) from January–December 2023 was performed for patients aged 0–64. Patients with the same diagnosis codes for the prior 12 months (i.e., chronic diagnosis), moderate to severe TBIs (S06.2–9), skull fractures (S02.xx), and/or brain hemorrhages (S06.3x) were excluded. **Results**: Out of 11,737,855 insured members with data in 2023, 43,213 new mTBIs were recorded (incidence rate = 0.37%), with the highest rate in adolescents (incidence rate = 1.27%). From the ages of 0–14 years, males had a higher incidence of concussion, but from 15 to 65 years, females had a higher incidence. Minimal differences were seen between urban and rural zip codes. **Conclusions**: Concussion incidence in adolescents is higher than other age groups, which may reflect increased participation in sports or heightened vulnerability during development. Males had a higher incidence than females during childhood, but females did later in life. These differences may reflect true disparities in injury risk, variations in reporting patterns, or a combination of both. Further research is warranted to understand the underlying mechanisms and to inform age- and sex-specific prevention efforts.

## 1. Introduction

Mild traumatic brain injuries (mTBIs) represent a major public health concern in the United States, affecting individuals across all demographics and activity levels [1]. Symptoms can be life-altering, affecting cognitive, social, emotional, and somatic domains, which may persist for weeks to months following the initial injury [2]. Epidemiological analyses estimate that millions of mTBIs occur annually [3], but true incidence is likely underreported [4,5]. This could be due to a variety of factors, including but not limited to individual attitudes, perceived self-efficacy, incomplete reporting, and differences in health-seeking behaviors and access to healthcare across populations [5,6]. Understanding the distribution of mTBI incidence across demographic subgroups is critical for both guiding prevention strategies and informing larger public health interventions.

The most used statistic for the incidence of mTBI in the United States (Langlois et al.) is over two decades old [7]. The authors estimate between 1.6 and 3.8 million traumatic brain injuries (TBIs) occurring every year, with the majority of them being mTBIs. This number was estimated using records of hospitalized injuries with loss of consciousness (LOC) and estimating the total incidence of mTBI while accounting that only 8–19.2% of these injuries present with LOC. More recent studies [8,9] have estimated the incidence of mTBI using more objective national hospitalization and insurance claims records; however, these are also over a decade old. Additional studies report important demographic patterns in mTBI risk, particularly by age [10,11] and sex [11,12]. Children and adolescents have the highest incidence [13]. School-aged males, particularly those from ages 9 to 12, have a higher rate of mTBI [14,15], while other studies report that females have a higher incidence in sex-comparable sports [16]. In addition to age and sex, examining the incidence and prevalence rates of mTBI based on area of residence is important, specifically in urban versus rural settings, to evaluate how geographic disparities shape distributions and outcomes. To our knowledge, no study has compared incidence rates of mTBI between rural and urban populations.

To address these gaps in knowledge, we utilized a nationwide insurance record registry to estimate the incidence of newly diagnosed mTBIs from January to December 2023 using International Classification of Diseases-10 (ICD-10) codes. We calculated the incidence of concussion by sex and year of life and by Center for Strategic and Budgetary Assessment (CSBA) stratification of rural and urban zip codes. We hypothesized that there would be a higher incidence of mTBI in adolescents, males, and in urban locations.

## 2. Materials and Methods

### 2.1. Study Design

This study was reviewed by the University of Buffalo’s Institutional Review Board (STUDY00010107) and was determined to be Exempt Non-Human Subject’s Research. The MarketScan^®^ Database was used for this study [17]. This database was originally developed by IBM Corporation and is currently owned by Truven Analytics. Stanford University’s Center for Population Health Sciences provides access to summarized pivot tables from this registry for outcomes research. The MarketScan^®^ Database includes person-specific clinical utilization, expenditures, and enrollment across inpatient, outpatient, prescription drug, and carve-out services for over 250 million persons from 2007 to 2023. The data comes from a selection of large employers and large private health insurance providers including BlueCross/BlueShield and Medicare/Medicaid.

### 2.2. Inclusion/Exclusion Criteria

A query using the ICD-10 codes of S06.0x.xx (mTBI) or F07.89 (post-concussion syndrome) from January–December 2023 was performed. To avoid including chronic injuries and to differentiate incidence from prevalence, we excluded patients who also had the same ICD-10 codes for the entire 12 months prior. Patients aged 0–64 years were included since data from patients aged 65 and above was considered a different data source (i.e., Medicare). This is described in the Limitations Section. Patients with moderate to severe TBIs (S06.2–9), skull fractures (S02.xx), and/or brain hemorrhages (S06.3x) were excluded. The ICD-10 code of S09.xx (unspecified head injury) is commented on in the discussion.

### 2.3. Analysis

Deidentified summary tables were provided by Accorded Actuarial Services, and no measures of variance (i.e., standard deviation or confidence intervals) were provided; therefore, no comparative statistical analysis could be carried out. CSBA defines rural zip codes as those with less than 2500 residents [18]. The incidence of injuries was calculated by dividing the number of new injuries by the number of insured patients for sex, year of life and rural/urban stratifications. Effect size (Cohen’s d) was calculated.

## 3. Results

From a total of 11,737,855 insured members with data in 2023, 43,213 new mTBIs were identified, corresponding to an overall incidence rate of 0.37% or 3.7 injuries per 1000 insured members per year. In 2023, the US population was 336.8 million [19], so this corresponds to 1.24 million diagnosed mTBIs every year in the US. If we hypothesize that half [7] of the mTBIs are not reported, then this estimate doubles to 2.48 million concussive injuries every year. Table 1 presents the insured sample size and number of injuries for each year of life from 0 to 64, stratified by patient sex.

**Figure 1:**
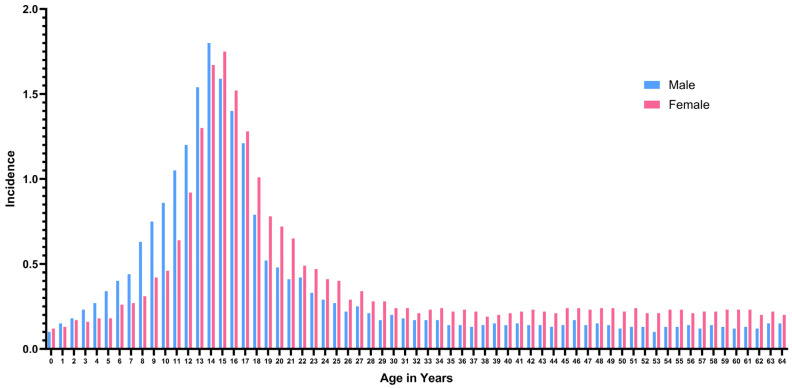
Percent incidence of concussions by sex and age of life.

Figure 1 presents the percent incidence of diagnosed mTBIs by year of life and sex. Males had a greater incidence before adolescence, whereas females had a greater incidence after adolescence.

Table 2 below presents the yearly percent incidence of mTBIs by age categories [20]. The yearly incidence of diagnosed mTBIs was 7 times higher in adolescents than older adults. When broken down by sex, males were 9.3 times more likely and females were 5.7 times more likely to be diagnosed with an mTBI in adolescence than in older age each year.

For our last aim, 9,987,519 records had zip codes that were stratified into rural and urban centers based on CSBA definitions. A total of 8,925,262 (89.36%) zip codes were from urban areas, whereas 1,062,257 (10.64%) zip codes were from rural areas. The incidence of mTBIs in rural areas was 0.384% and in urban areas it was 0.420%. A difference of 0.036% in incidence of mTBIs between rural and urban areas suggests minimal differences (Cohen’s d = 0.0001).

## 4. Discussion

This study reports the incidence of mTBIs in insured US-persons from age 0–64 by sex and by rural–urban classification. The estimated incidence of mTBIs for all people was 0.37% per year. Using Census.gov (https://www.census.gov/) population statistics from 2023 [20], it can be estimated that there were 1.24 million diagnosed mTBIs in the US in 2023. These findings are in line with the primary hypothesis: mTBI incidence is higher in the adolescent age group. This has been reported in several previous publications [21]. Proposed mechanisms include higher vulnerability of the developing brain, weaker neck muscles, and larger head-to-body ratios [21]. Children and adolescents participate in contact sports more than adults, which is a leading cause of mTBIs worldwide [22]. The incidence of mTBIs decreased in college-aged students compared to high school-aged students, even though college students also participate in organized sports. This may be an underestimation since colleges with National Collegiate Athletic Association intercollegiate athletics have in-house sports medicine physicians for treatment that do not utilize standard insurance billing (i.e., these are salaried physicians). These injuries could not be included in our study design, which may explain why college-aged people had a lower incidence than expected.

The second hypothesis was not consistent with our findings. We hypothesized that males would have a greater incidence of mTBI throughout life, but this was seen only for the 0–14-year cohort. In the 15–64-year cohort, females had a higher incidence of diagnosed mTBI. Although most publications [13] report a higher incidence of mTBI in males, a study [23] using data from the National Electronic Injury Surveillance System which stratified by age groups reported that males under the age of 25 sustained mTBIs more often, whereas females had a greater incidence after the age of 25. The current study that stratifies by every year of life found that this shift occurs around the age of 15. It is unknown whether females in this dataset sustained more mTBIs than males or if this represents a difference in reporting behavior. It is known that women disproportionally receive more traumatic injuries due to intimate partner violence; however, most of these injuries go untreated and unreported [24] and theoretically should not be a contributing factor for females having a higher number of diagnosed mTBIs, specifically in data from insurance-reimbursed healthcare services.

Regarding the third hypothesis, the incidence difference between rural and urban areas was negligible, but only ~10% of our data came from rural zip codes. In 2020, 20.0% of the US lived in rural zip codes, which is double of what we observed in our sample. It is unknown why this discrepancy was observed. It may be due to people residing in rural areas having less access to healthcare or having less injuries. Our study design cannot explain the rationale for why patients seek healthcare services, which limits our interpretation, but it can be assumed that rural areas are underrepresented in healthcare research and future studies should be conducted to understand these differences.

The incidence of mTBIs in this dataset is likely a substantial understatement of the true incidence of mTBIs. Up to half of the mTBIs are not reported [7], especially in a sports setting [25], and cannot be identified by screening health insurance claims records. If we include these, then our estimated incidence will double to 2.48 million injuries a year and be more similar to incidences reported by Langlois et al. [7]. One reason for underreporting is that signs and symptoms of mTBI vary, with some having mild symptoms and others experiencing debilitating symptoms [26]. This may influence a person’s likelihood to seek care, since those with milder symptoms may delay seeking treatment until they realize that their symptoms are not improving [27]. Unless the injury is observed during an organized or professional sport setting, it is the patient’s responsibility to seek care themselves. This can be affected by a variety of factors, such as individual health-seeking behaviors or access to healthcare [28]. Athletes may tend to hide their injuries in fear of missing out on a sport, which is another factor that contributes to the underreporting of mTBIs. Another major reason for underestimation is that we only searched for mTBI or post-concussion syndrome ICD-10 codes, but mTBIs can be classified as unspecified head injuries (S09.xx series ICD-10 codes) in the emergency department. It is estimated that 50–58% [29,30,31] of head injuries are coded as S09.xx in the emergency department, and another study reports that the positive predictive value of S09 for mTBIs is 36–52% [32]. If this is accounted for, then we expect our estimate to increase by another 50%. We were financially limited in procuring more data for the current study, but a future investigation of the S09.xx ICD-10 codes is warranted.

## 5. Limitations

While this study provides insight into mTBI incidence, there are several limitations to consider. The authors did not have access to the raw data and were dependent on a commercial company to provide summarized reports which may have been filtered versions of the source data. We also did not have information about mTBI severity or their clinical consequences. Future investigations should consider collecting clinical mTBI data in addition to incidence data to determine how reporting behavior may be impacted by injury severity, symptom burden, and recovery trajectories. Another limitation is that we did not include data on patients aged 65 and older due to the additional cost for this data and funding limitations. Excluding this population may not account for those who need the most care and have the most substantial burden on healthcare due to their comorbidities. Another limitation is that even though we used a database of 12 million people, this only represents about 3.7% of the US population in that year, which can raise the risk of Type I and II statistical errors. Our study design could not account for people without insurance, which accounts for 9.1% of the US population in 2023 [20]. Future research should include patients without insurance and those 65 and older, and it should further investigate the mechanisms behind the differences seen in our dataset.

## 6. Conclusions

The estimated incidence of diagnosed mTBIs or concussions for all people in the US is 3.7 injuries per 1000 persons per year, or 1.24 million mTBIs per year. The incidence of mTBIs in adolescents is higher than that in other age groups, which may reflect increased participation in sports or heightened vulnerability during development. Males had a greater incidence than females during childhood, but females had higher rates from adolescence into adulthood. These differences may reflect true disparities in injury risk, variations in reporting patterns, or a combination of both. Minimal differences in incidence were observed between rural and urban areas. Further research is warranted to understand the underlying mechanisms and to inform age- and sex-specific prevention efforts.

## Figures and Tables

**Table 1:** Number of concussions and sample size by sex and age of life.

Age	Male mTBI	Male Sample Size	Female mTBI	Female Sample Size	Age	Male mTBI	Male Sample Size	Female mTBI	Female Sample Size
0	63	63,839	73	61,360	33	159	91,450	236	100,600
1	97	64,714	82	61,525	34	152	91,795	238	100,926
2	114	63,714	107	61,291	35	131	91,880	226	101,277
3	156	67,613	106	64,667	36	132	92,083	230	101,406
4	185	68,989	117	66,795	37	122	93,930	232	103,311
5	244	71,897	127	68,918	38	129	94,636	195	105,307
6	303	74,920	183	71,704	39	140	93,450	203	103,280
7	325	74,152	195	71,050	40	136	95,203	224	106,845
8	496	78,648	238	75,580	41	144	96,839	232	107,863
9	603	80,569	323	76,604	42	136	96,265	245	107,903
10	707	81,866	362	79,232	43	138	95,752	237	107,402
11	872	83,064	512	79,609	44	119	94,095	218	105,757
12	1022	85,050	750	81,791	45	133	92,883	256	104,662
13	1352	87,630	1097	84,463	46	165	94,585	255	105,608
14	1649	91,407	1464	87,899	47	129	91,156	237	101,801
15	1490	93,754	1587	90,792	48	143	94,571	255	105,254
16	1342	95,529	1398	91,994	49	133	94,176	248	103,251
17	616	50,746	629	49,103	50	116	95,888	234	105,932
18	750	94,943	920	91,435	51	130	100,524	268	110,359
19	500	95,823	722	92,476	52	138	107,329	243	118,342
20	465	96,181	670	92,906	53	106	104,879	244	114,857
21	393	95,459	600	91,997	54	135	100,908	256	111,088
22	413	98,369	479	96,897	55	130	97,488	243	106,923
23	340	102,696	477	101,751	56	133	97,692	224	108,056
24	301	103,992	422	103,265	57	118	98,463	240	108,656
25	217	80,066	321	80,913	58	144	102,254	245	112,424
26	130	58,267	180	61,545	59	131	103,660	270	114,913
27	166	66,286	240	71,300	60	121	102,656	264	113,175
28	148	71,578	216	77,420	61	128	99,920	252	110,580
29	131	76,208	232	82,197	62	117	97,685	219	108,901
30	159	81,156	212	87,350	63	143	93,929	223	103,365
31	150	84,017	218	92,295	64	72	49,519	107	53,523
32	146	87,992	207	97,507	Total	20,248	5,718,677	22,965	6,019,178

**Table 2:** Yearly percent incidence of mTBIs by age categories.

Age Groups	Male	Female
Infant and Toddler (0–2 years)	0.143%	0.142%
Childhood (3–11 years)	0.571%	0.331%
Adolescent (12–19 years)	1.255%	1.279%
Young Adult (20–39 years)	0.235%	0.310%
Older Adult (40–64 years)	0.135%	0.223%

## Data Availability

Data are contained within the article.

## Abbreviations

The following abbreviations are used in this manuscript:

mTBI	Mild traumatic brain injury
TBI	Traumatic brain injury
LOC	Loss of consciousness
CSBA	Center for Strategic and Budgetary Assessment
US	United States
ICD	International Classification of Diseases
